# Supplementary material for: Evaluation of Nonresponse Bias in a Case–Control Study of Pleural Mesothelioma
Source: Int J Environ Res Public Health. 2020 Aug 24;17(17):6146. doi: 10.3390/ijerph17176146 (PMC7504238; doi:10.3390/ijerph17176146)
Supplement: Supplementary file 1 [file ijerph-17-06146-s001.pdf]

Supplementary Materials:

Table S1. Description of responder controls for levels of education, occupation and marital status in different administrative databases. The number of subjects and the percentage are reported.

Variables	Registrar Office		HAR (Subjects With At Least One Hospitalization)		Questionnaire	
	n	%	*n*	%	*n*	%
**Educational level**						
Primary school	70	40.2	20	16.7	74	42.5
Middle school	26	14.9	16	13.3	44	25.3
Secondary school	31	17.8	16	13.3	38	21.8
University	4	2.3	3	2.5	9	5.2
Missing	43	24.7	65	54.2	9	5.2
	174	100	120	100	174	100
**Occupation**						
Employed	48	27.6	25	20.8	73	41.9
Unempoloyed	17	9.8	9	7.5	26	14.9
Retired	81	46.5	49	40.8	75	43.1
Missing	28	16.1	37	30.8	0	0.0
	174	100	120	100	174	100
**Civil status**						
Married/cohabitant	106	60.9	76	63.3	130	74.7
Widowed	38	21.8	10	8.3	24	13.8
Unmarried	15	8.6	12	10.0	16	9.2
Separated/divorced	5	2.9	3	2.5	4	2.3
Missing	10	5.7	19	15.8	0	0.0
	174	100	120	100	174	100

Table S2. Cohen's kappa for education, occupation and civil status comparing Hospital Admission Records (HAR) and Town Registrar's records (TR) to Questionnaire data.

**Sources**	**Variables**	**Questionnaire**					
	**Educational Level**	Low	High		Missing	Total	Kappa
HAR	low	33	2		1	36	0.7051
	high	5	14		0	19	
	missing	48	14		3	65	
	Total	86	30		4	120	
	**Occupation**	employed	not employed	retired	missing	Total	kappa
HAR	employed	22	0	3	0	25	0.5000
	not employed	3	5	1	0	9	
	retired	12	6	31	0	49	
	missing	9	19	9	0	37	
	Total	46	30	44	0	120	
	**Civil status**	Married/ cohabitant	Widowed/ separated	unmarried	missing	Total	kappa
HAR	married/cohabitant	69	7	0	0	76	0.7180
	widowed /Separated	2	11	0	0	13	
	unmarried	2	1	9	0	12	
	missing	14	3	2	0	19	
	Total	87	22	11	0	120	
**Sources**	**Variables**	**Questionnaire**					
	**Educational level**	low	high		missing	Total	kappa
TR	low	85	6		5	96	0.8266
	high	3	32		0	35	
	missing	30	9		4	43	
	Total	118	47		9	174	
	**Occupation**	employed	not employed	retired	missing	Total	kappa
TR	employed	39	0	9	0	48	0.5013
	not employed	5	11	1	0	17	
	retired	20	9	52	0	81	
	missing	9	6	13	0	28	
	Total	73	26	75	0	174	
	**Civil status**	Married/ cohabitant	Widowed/ separated	unmarried	missing	Total	kappa
TR	married/cohabitant	106	0	0	0	106	0.7629
	widowed /Separated	18	25	0	0	43	
	unmarried	0	0	15	0	15	
	missing	6	3	1	0	10	
	Total	130	28	16	0	174	
